# Supplementary material for: Preclinical Assessment in Transgenic NOD Mice of a Novel Immunotherapy for Type 1 Diabetes: Lipoplexes Down-Modulate the Murine C1858T Ptpn22 Variant In Vitro
Source: Int J Mol Sci. 2025 Nov 21;26(23):11241. doi: 10.3390/ijms262311241 (PMC12692099; doi:10.3390/ijms262311241)

Supplementary Figure 1: Percentage (%) of inhibition 48 and 72h post-treatment with Lipofectamine (**A**, **B**) and LiposiRNA (**C**, **D**).

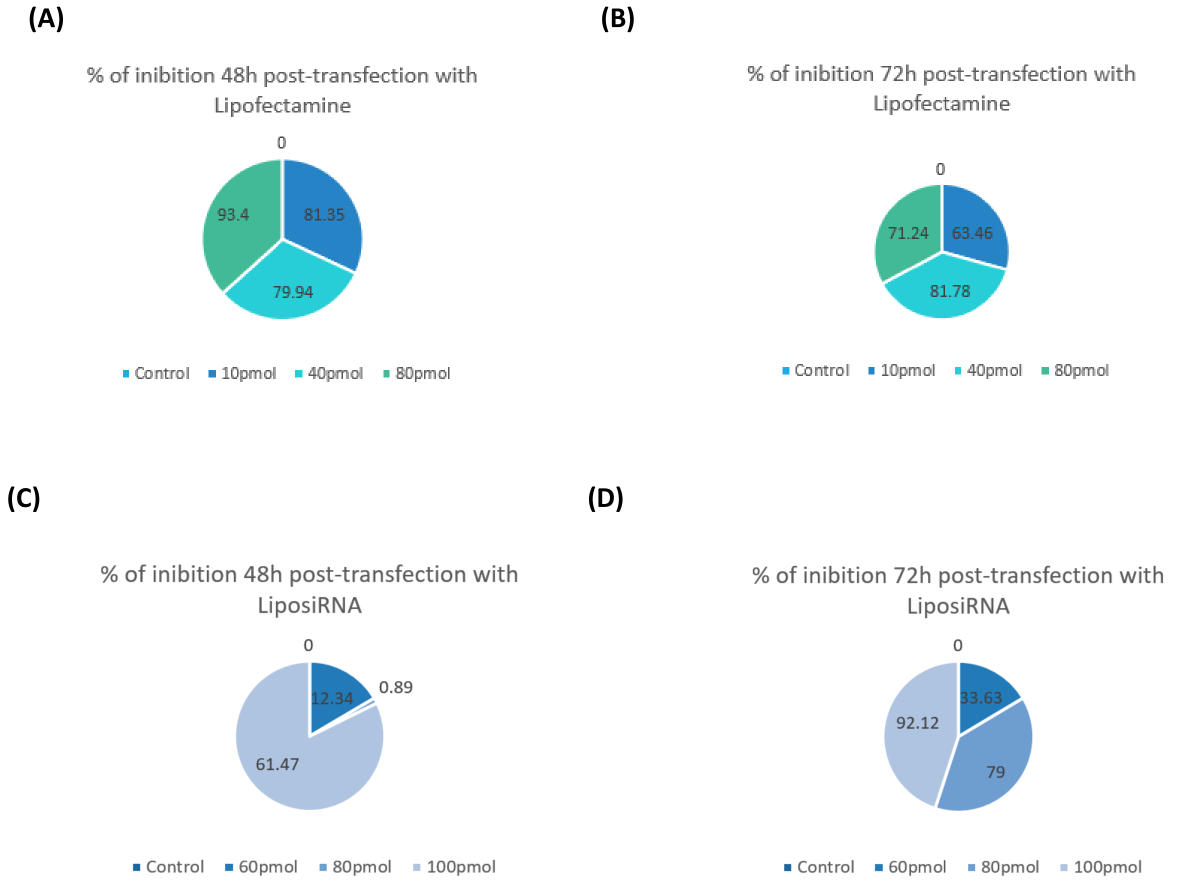

Supplement: Supplementary file 1 [file ijms-26-11241-s001.zip › Supplementary Figure S1.pdf]
