# Supplementary material for: Preclinical Assessment in Transgenic NOD Mice of a Novel Immunotherapy for Type 1 Diabetes: Lipoplexes Down-Modulate the Murine C1858T Ptpn22 Variant In Vitro
Source: Int J Mol Sci. 2025 Nov 21;26(23):11241. doi: 10.3390/ijms262311241 (PMC12692099; doi:10.3390/ijms262311241)
Supplement: Supplementary file 1 [file ijms-26-11241-s001.zip › Supplementary Table S1.pdf]

Supplementary Table 1: Comprehensive statistical results for Figures 1 and 2.

48h post-transfection with Lipofectamine				
	**Control**	**10pmol**	**40pmol**	**80pmol**
mean ± SD	*1 ± 0*	*0.1865 ± 0.1059*	*0.2006 ± 0.0928*	*0.0660 ± 0.0186*
**F(DFn, DFd)** $F(3, 4) = 72.86$ **Pvalue** $P = 0.0006$				

72h post-transfection with Lipofectamine				
	**Control**	**10pmol**	**40pmol**	**80pmol**
mean ± SD	*1 ± 0*	*0.3654 ± 0.0423*	*0.1822 ± 0.0928*	*0.2876 ± 0.0452*
**F(DFn, DFd)** $F(3, 4)= 87.12$ **Pvalue** $P=0.0004$				

48h post-transfection with LipoSirna				
	**Control**	**60pmol**	**80pmol**	**100pmol**
mean	*1 ± 0*	*0.8766 ± 0.3131*	*0.9911 ± 0.0126*	*0.3853 ± 0.0226*
**F(DFn, DFd)** $F(3, 4) = 6.849$ **Pvalue** $P = 0.0470$				

72h post-transfection with LipoSirna				
	**Control**	**60pmol**	**80pmol**	**100pmol**
mean	*1 ± 0*	*0.6637 ± 0.2447*	*0.2100 ± 0.1428*	*0.0788 ± 0.0150*
**F(DFn, DFd)** $F(3, 4) = 17.81$ **Pvalue** $P = 0.0089$				
